# CDC5L drives FAH expression to promote metabolic reprogramming in melanoma

**DOI:** 10.18632/oncotarget.23107

**Published:** 2017-12-07

**Authors:** Zhichao Gu, Huafeng Zhang, Yong Li, Susu Shen, Xiaonan Yin, Wei Zhang, Ruimin Cheng, Yong Zhang, Xiaoyan Zhang, Hui Chen, Bo Huang, Yuchun Cao

**Affiliations:** ^1^ Department of Dermatology, Tongji Hospital, Tongji Medical College, Huazhong University of Science and Technology, Wuhan 430030, China; ^2^ Department of Biochemistry and Molecular Biology, Tongji Medical College, Huazhong University of Science and Technology, Wuhan 430030, China; ^3^ Department of Oncology, Renmin Hospital, Hubei University of Medicine, Shiyan, Hubei 442000, China; ^4^ National Key Laboratory of Medical Molecular Biology and Department of Immunology, Institute of Basic Medical Sciences, Chinese Academy of Medical Sciences, Beijing 100005, China

**Keywords:** fumarylacetoacetate hydrolase (FAH), anaplerotic reactions, cell division cycle 5-like protein (CDC5L), tumor metabolic reprogramming, melanoma

## Abstract

Metabolic reprogramming allows tumor cells to thrive in the typically hypoxic tumor microenvironment. Using immunodetection and clinical data analyses, we demonstrate here that fumarylacetoacetate hydrolase (FAH) is highly expressed in melanoma and correlates with poor survival. FAH knockdown inhibits proliferation and migration, while promoting apoptosis in melanoma cells, result in prolonged survival in tumor-bearing mice. Molecular analyses using real time RT-PCR, western blot, and ^13^C tracing showed that these changes are driven by strong stimulation of anaplerotic reactions through the TCA cycle and the pentose-phosphate pathway, resulting in increased fatty acid and nucleotide synthesis. Using bioinformatic, ChIP-PCR, and gene silencing analyses, we determined that cell division cycle 5-like protein (CDC5L) is an important transcription factor regulating FAH expression in melanoma cells. These findings reveal that FAH induces metabolic reprogramming in melanoma and so emerges as both a potentially useful independent prognostic indicator and an attractive therapeutic target.

## INTRODUCTION

Melanoma, a malignant tumor that arises from melanocytes, is the most dangerous skin tumor, displaying high recurrence and a low survival rate that contribute to more than 50,000 deaths per year worldwide. Most researchers pinpoint excessive ultraviolet light exposure as the primary cause of melanoma, especially in people with low skin pigmentation. Current treatments include surgical removal, chemotherapy, radiotherapy, and immunotherapy, which are, especially at advanced tumor stages, characterized by poor efficacy. Therefore, there is an urgent need to define new therapeutic targets and prognostic indicators for this disease.

Increasing evidence suggests that metabolic reprogramming is a critical hallmark of tumor cells [1–3]. This phenomenon is considered to be largely regulated by oncogenic activation, which might be linked to specific transcription factor activity, signal transduction pathways, and/or epigenetic mechanisms [4]. Unlike normal cells, which have a comparatively low rate of glycolysis and derive their energy mainly from oxidation of pyruvate in the mitochondria, most cancer cells produce energy predominantly by enhanced glycolysis in the cytoplasm, even if oxygen is plentiful (i.e., the Warburg effect) [5–8]. Initially, the Warburg effect was thought to be the result of mitochondrial damage or shut-down mediated by cancer genes or other mechanisms [9]. However, mounting evidence demonstrated that mitochondrial functions, albeit frequently dysregulated in cancer cells, play essential biosynthetic roles in various tumors, especially in melanoma, and contribute to metabolic reprogramming by enhancing anaplerotic reactions [10–13]. Anaplerotic reactions are chemical reactions that form biosynthetic intermediates within metabolic pathways such as the tricarboxylic acid (TCA) cycle. In the TCA cycle, concentrations of intermediates typically remain constant despite multiple biosynthetic reactions that utilize these molecules as substrates. Anaplerosis refers to the process of replenishing TCA cycle intermediates that have been extracted for biosynthesis through cataplerotic reactions [1, 13]. Four main reactions, catalyzed by pyruvate carboxylase (PC), aspartate transaminase, glutamate-dehydrogenase, methylmalonyl-CoA mutase, and adenylosuccinate lyase, have been described during anaplerosis [1]. The most important one among these is considered to be the irreversible carboxylation of pyruvate into oxaloacetate by PC [14–16]. Anaplerotic flux must balance cataplerotic flux to maintain the homeostasis of multiple metabolic pathways, such as the TCA cycle, in normal tissues and in tumors such as melanoma [13, 17].

Fumarylacetoacetate hydrolase (fumarylacetoacetase; FAH) is an enzyme encoded by the *FAH* gene, which is located on chromosome 15q25.1 and contains 14 exons. FAH is the last enzyme in the sub-pathway of L-phenylalanine and tyrosine degradation, catalyzing the hydrolysis of 4-fumarylacetoacetate into fumarate and acetoacetate [18, 19]. Mutations in the *FAH* gene cause type I tyrosinemia [20], a hereditary metabolic condition characterized by increased tyrosine levels in the blood and urine of patients [21]. Once produced, fumarate can be converted to malate by fumarase (fumarate hydratase; FH) in the cytoplasm. Then, through transporters such as SLC25A1, SLC25A10, and SLC25A11 [22], or via citrate-malate antiport, malate may be directly transported into the mitochondria to participate in the TCA cycle. Alternatively, malate may be converted to pyruvate by malic enzyme (ME). Pyruvate then enters the mitochondria and is converted to oxaloacetate (OAA) via PC or to acetyl-CoA by the pyruvate dehydrogenase complex (PDC), to fuel the TCA cycle.

Whether FAH participates in anaplerotic reactions in melanoma cells remains unclear. In the present study, we evaluated FAH expression in clinical samples, conducted survival analyses in both patients’ data and a mouse melanoma model, and performed bioinformatics, real time RT-PCR, western blot and ^13^C tracing analyses to assess the role of FAH in human melanoma cells. Our results indicate that FAH transcription, driven by cell division cycle 5-like protein (CDC5L), is essential in the metabolic reprogramming of melanoma by promoting important anaplerotic reactions that sustain tumor growth and potentiate the disease’s severity.

## RESULTS

### FAH is highly expressed in melanoma and correlates with poor clinical outcomes

**Figure 1:**
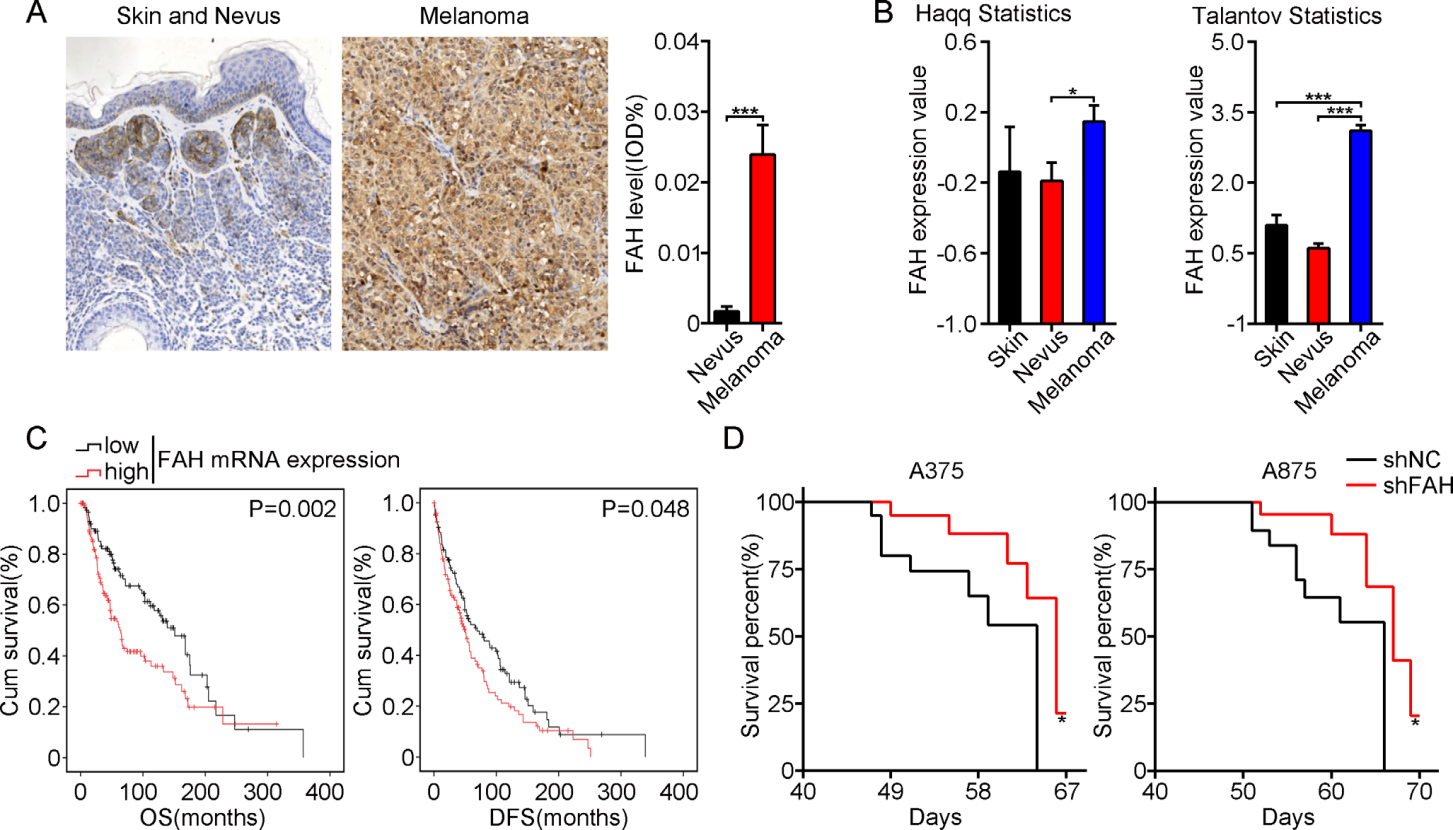
FAH expression is inversely associated with survival in both melanoma patients and melanoma-bearing mice (**A**) FAH expression by IHC in representative samples of a human melanoma tissue microarray. Data are expressed as mean IOD values; error bars represent SEM; ^***^p < 0.001 (Student’s *t-test*). (**B**) FAH mRNA expression in melanoma, nevus, and skin tissues from two different datasets (Haqq and Talantov) analyzed using the Oncomine database. Error bars indicate SEM; ^*^p < 0.05; ^***^p < 0.001 (Student’s *t-test*). (**C**) FAH mRNA and survival information from 278 skin melanoma patients (TCGA database). D. Survival analysis of BALB/c-nude mice inoculated with melanoma cells stably expressing shRNA directed against FAH. Data are mean ± SEM; *n* = 8; ^*^p < 0.05; ^***^p < 0.001 (Student’s *t-test*).

Analysis of a human melanoma tissue microarray by immunohistochemistry (IHC) with a FAH antibody revealed significantly higher, mainly cytosolic, FAH expression in melanoma than in benign nevus and normal skin (Figure 1A, Supplementary Figure 1, and Supplementary Table 1). Consistently, the same results were found in two different melanoma registries (Haqq and Talantov) listed in the Oncomine database (Figure 1B). To assess whether FAH levels correlate with clinical outcomes in melanoma patients, we obtained detailed FAH mRNA levels and survival information from 278 melanoma patients from TCGA Research Network (http://cancergenome.nih.gov/). The association between FAH levels and overall survival (OS) and disease-free survival (DFS) was estimated by Kaplan-Meier survival analysis. Data showed that patients with high FAH levels exhibited significantly shortened OS and DFS than those in which FAH expression was low (Figure 1C), suggesting the potential of FAH as an independent prognostic indicator for melanoma.

To model and test this association, we transduced human A375 and A875 melanoma cells with lentivirus expressing shRNA against FAH (shFAH) or scrambled (control) shRNA and then performed puromycin selection to establish stable clones. Successful construction of the lentiviral vectors was confirmed by direct nucleic acid sequencing (data not shown). FAH expression was confirmed by GFP detection using fluorescence microscopy, real-time RT-PCR, and western blot (Supplementary Figure 2A–2C). A tumor-bearing mouse model was then established by injecting shFAH-A375 or shFAH-A875 cells into the flanks of nude mice, and survival times were recorded. Consistent with the observations in patients, the survival time of FAH-silenced animals was longer than that of the control groups (Figure 1D). These results showed that high FAH expression is common in melanoma and is associated with shortened survival times in both patients and tumor-bearing mice.

### FAH expression promotes proliferation, migration, and survival in melanoma cells

**Figure 2:**
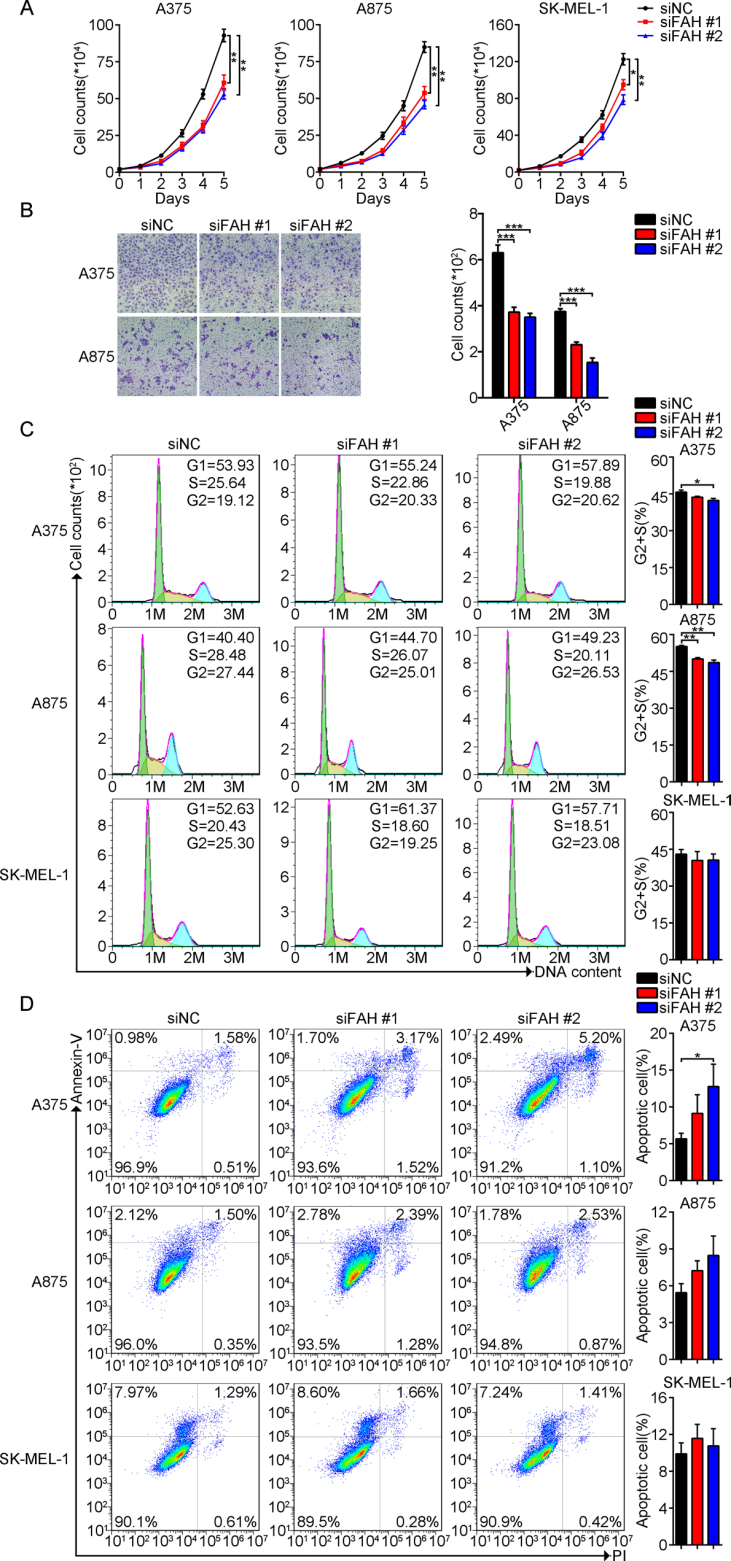
FAH knockdown arrests cell cycle, reduces proliferation and migration, and increases apoptosis in melanoma cells (**A**) Proliferation assay in melanoma cells transfected with FAH siRNA or scrambled siRNA and cultured in complete medium. Cell counts were obtained at different time points. (**B**) Transwell migration assay in melanoma cells transfected with FAH siRNA or scrambled siRNA. (**C–D**) Flow cytometric evaluation of cell cycle staging and apoptosis in melanoma cells transfected with FAH siRNA or scrambled siRNA. Data from 3 independent experiments are expressed as mean ± SEM; ^*^p < 0.05; ^**^p < 0.01; ^***^p < 0.001 (Student’s *t-test*).

To investigate the impact of FAH fluctuations on melanoma cell growth and survival, we first knocked down FAH expression using two different siRNAs and analyzed proliferation, migration, apoptosis, and cell cycle staging. The silencing efficiency of both siRNAs was verified using real-time RT-PCR and western blot (Supplementary Figure 2D). Compared with the respective control groups, both proliferation and migration were significantly decreased in FAH-silenced A375, A875, and SK-MEL-1 cells, and these effects correlated with FAH silencing efficiency (Figure 2A–2B). Interestingly, FAH knockdown or overexpression did not influence A375 cell proliferation in serum-free medium, suggesting that impaired migration does not result from decreased proliferation (Supplementary Figure 2E). We also detected the expression of several important proteins involved in epithelial-mesenchymal transition (EMT) in melanoma cells. For these, no noticeable expression changes were seen after FAH knockdown (Supplementary Figure 2F). We next evaluated the consequences of FAH silencing on cell cycle staging and apoptosis using flow cytometry. Results showed that in the melanoma cells the two siFAHs tested effectively reduced the number of cells in G2+S, while increasing the number of cells in G1 (Figure 2C). Meanwhile, Annexin V-FITC staining experiments showed that FAH knockdown was associated with increased apoptosis in the melanoma cell lines (Figure 2D).

Next, we increased the expression of FAH in melanoma cells using a plasmid vector (FAH-pcDNA3.1). Direct DNA sequencing (data not shown) and agarose gel electrophoresis (Supplementary Figure 2G) were used to validate the construct. Transfection efficiency was verified by real-time RT-PCR and western blot (Supplementary Figure 2G). FAH-pcDNA3.1 transfection did not affect proliferation in melanoma cells (Supplementary Figure 3A) but increased, as expected, their rate of migration compared with control cells (Supplementary Figure 3B). Cell cycle analysis showed a decreased G1 population, and an increase in the number of cells in G2+S phase (Supplementary Figure 3C), while no noticeable differences in the rate of apoptosis were observed between the FAH-overexpressing and control cells (Supplementary Figure 3D). We speculated that this phenomenon might be associated with sufficient endogenous FAH expression and limited substrates in melanoma cells. Thus, combined results from FAH silencing and overexpression experiments suggest that FAH promotes proliferation and migration, while inhibiting apoptosis in melanoma cells.

### Analysis of FAH-regulated gene expression in melanoma cells

**Figure 3:**
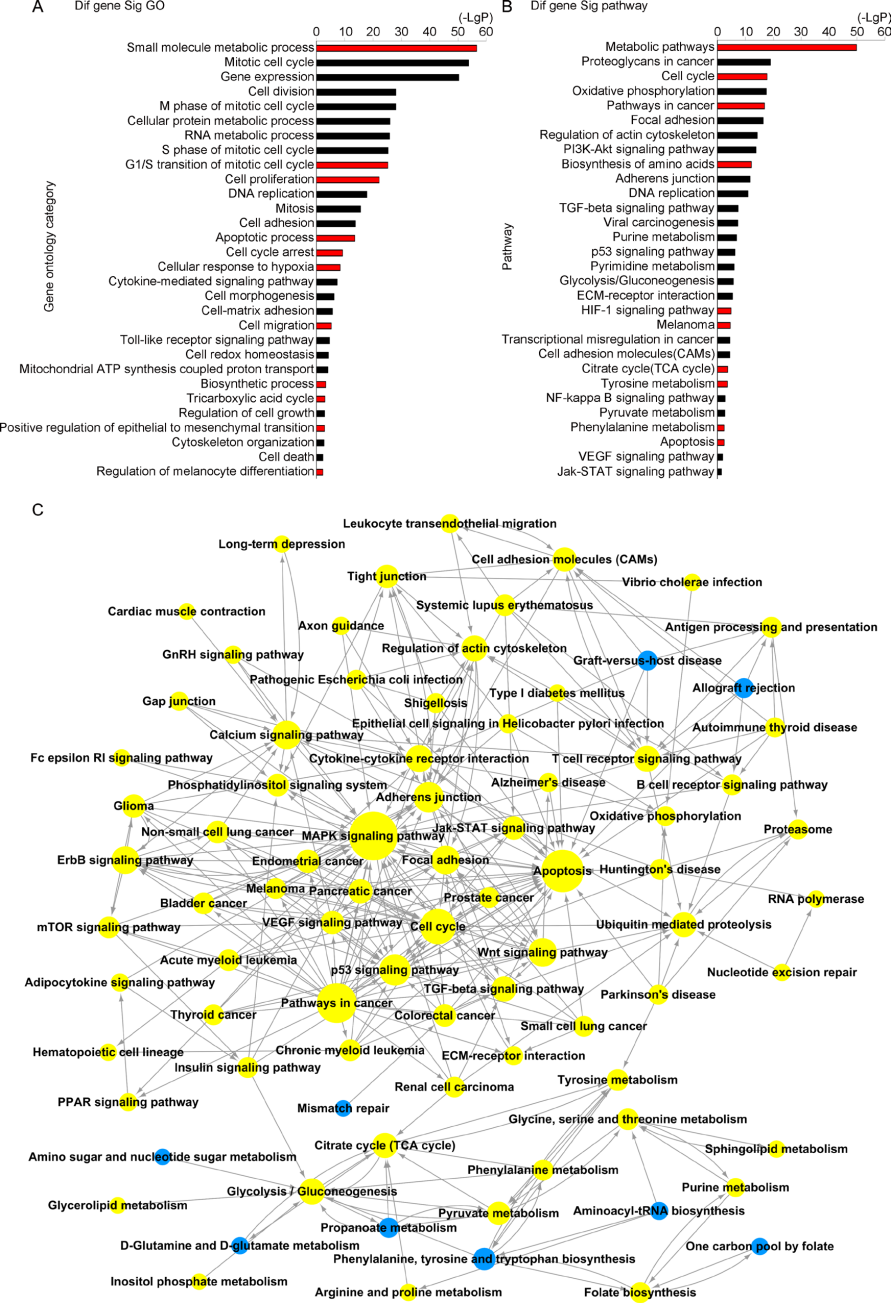
GO and pathway interaction analyses (**A–B**) GO and pathway analyses of differentially expressed genes (DEGs) in A375 cells after siRNA-mediated FAH silencing. GO analysis was applied to analyze the DEGs’ main functions. Pathway analysis was predominantly based on the KEGG database. The vertical axis represents categories and the horizontal axis represents -log 10 *p* values of significant GO terms or pathways. (**C**) Pathway network (PathNet) was constructed by connecting significant pathways including DEGs identified upon FAH knockdown. Circles represent pathways and lines indicate pathway interactions.

To shed light on the biological processes regulated by FAH, we performed mRNA expression profiling in control and FAH-knockdown melanoma cells. A375 cells were transfected in triplicate with siFAH or a scrambled siRNA for 72 h. Microarray analysis using Affymetrix GeneChip Human Gene 1.0 ST arrays identified 2535 DEGs after FAH silencing (Supplementary Table 2). Gene Ontology (GO) analysis was next employed to interrogate the biological functions of these DEGs. Significantly enriched GO terms were clustered based on their functional annotations. The data showed that for these 2535 DEGs, there were 512 functionally annotated clusters that were significantly enriched. The main clusters were involved in small molecule metabolic process, G1/S transition of cell cycle, cell proliferation, cellular response to hypoxia, cell migration, biosynthetic process, TCA cycle, and cell death (Figure 3A). Based on the Kyoto Encyclopedia of Genes and Genomes (KEGG) database, we further analyzed the pathways in which the DEGs were significantly enriched by applying the PathNet algorithm [23] (Figure 3B–3C). In line with GO analysis results, DEG involvement was detected in 164 pathways, affecting cell cycle, apoptosis, the TCA cycle, tyrosine metabolism, and HIF-1 signaling, among others. Overall, these results suggest that FAH may affect multiple important biological functions and metabolic processes, including the TCA cycle, in melanoma cells.

### FAH promotes anaplerotic reactions and fatty acids synthesis in melanoma cells

**Figure 4:**
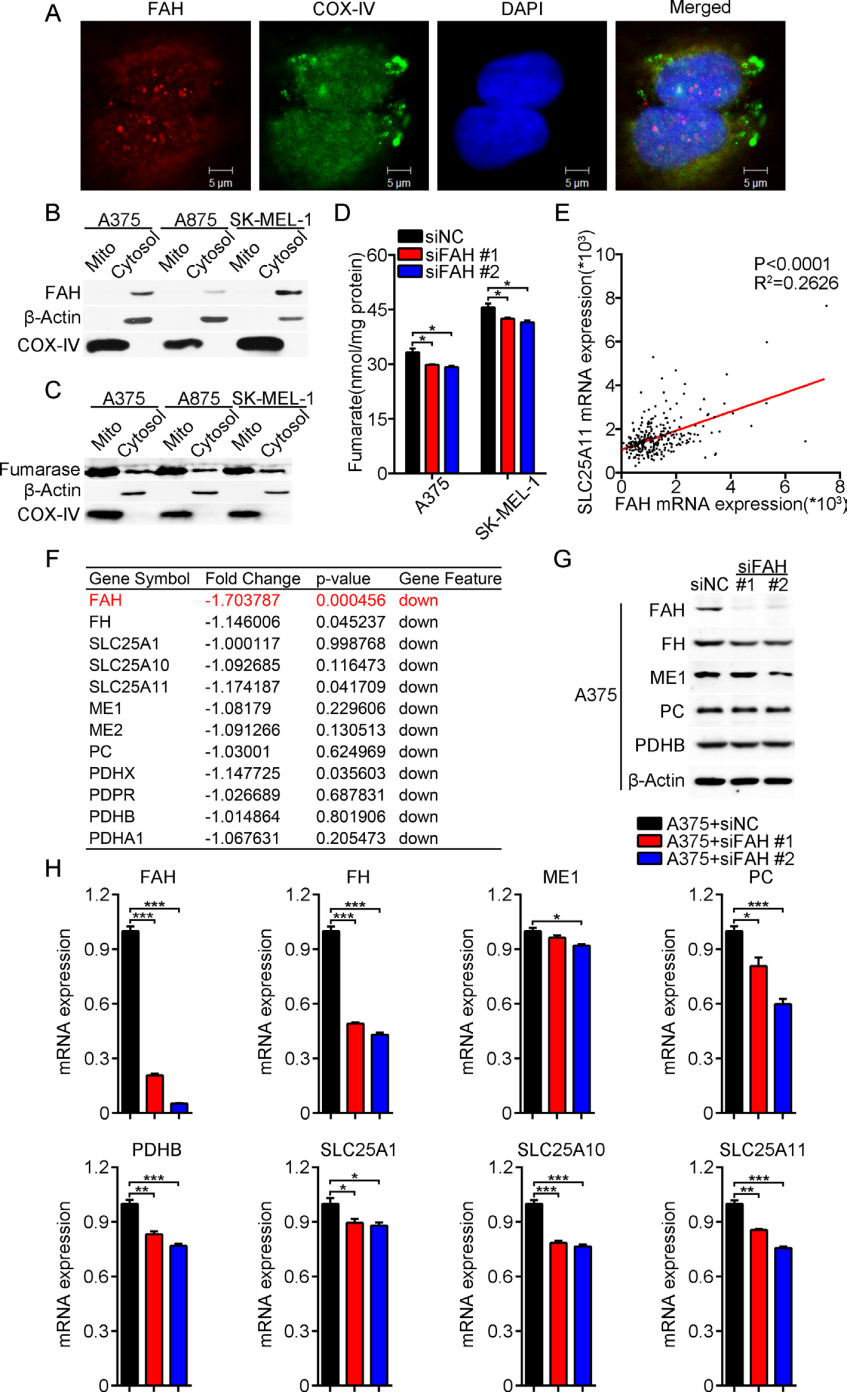
FAH expression modulates TCA cycle enzyme and transport activity (**A**) A375 cells were stained with FAH or COX-IV antibodies and photographed under a two-photon confocal microscope. Scale bars, 5 μm. (**B**) Western blot analysis of FAH expression in cytosolic and mitochondrial fractions isolated from A375, A875, and SK-MEL-1 melanoma cells. β-actin and COX-IV served as cytosolic and mitochondrial markers, respectively. (**C**) Western blot analysis of β-actin, COX-IV, and fumarase in cytosolic and mitochondrial fractions of A375, A875, and SK-MEL-1 melanoma cells. (**D**) Total cellular fumarate levels in siFAH-transfected melanoma cells. (**E**) FAH and SLC25A11 mRNA data from clinical samples of melanoma (TCGA database). (**F**) Expression profiling of FAH, FH, SLC25A1, SLC25A10, SLC25A11, ME1, ME2, PC, PDHX, PDPR, PDHB, and PDHA1 mRNAs in FAH-silenced A375 cells. (**G**) Western blot analysis of FAH, FH, ME1, PC, and PDHB in siFAH-transfected A375 cells. (**H**) Real-time RT-PCR analysis of FAH, FH, ME1, PC, PDHB, SLC25A1, SLC25A10, and SLC25A11 mRNAs in siFAH-transfected A375 cells. Data are presented as mean ± SEM; *n* = 3. ^*^p < 0.05; ^**^p < 0.01; ^***^p < 0.001 (Student’s *t-test*).

FAH synthesizes fumarate and acetoacetate from L-phenylalanine and tyrosine. In the melanoma cells studied here, both immunostaining and western blot showed that FAH localized mainly in the cytosol, rather than the mitochondria (Figure 4A–4B). We speculated that fumarate formed by FAH activity is converted to malate by fumarase (FH), which is then transported into the mitochondria, where it fuels anaplerotic reactions via the TCA cycle. To assess this hypothesis, we first analyzed the expression of fumarase in melanoma cells. Consistent with previous reports [24], western blots showed both cytosolic and mitochondrial fumarase expression (Figure 4C). On the other hand, and as expected, fumarate levels decreased after FAH knockdown (Figure 4D) and increased after ectopic FAH expression (Supplementary Figure 4A). Then, we accessed the cBioPortal for Cancer Genomics [25, 26] to evaluate and correlate mRNA levels of FAH and the oxoglutarate/malate carrier SLC25A11 in cutaneous melanoma data from TCGA. Results showed that indeed, the mRNA levels of FAH were positively correlated with those of SLC25A11 (Figure 4E).

Next, we performed mRNA expression profiling in FAH-silenced A375 cells, and found that the expression of multiple enzymes, including FH, malic enzyme (ME1 and ME2), pyruvate dehydrogenase complex (PDHB, PDHX, PDPR, and PDHA1), pyruvate carboxylase (PC), and the mitochondrial carrier proteins SLC25A1, SLC25A10, and SLC25A11, was downregulated after FAH knockdown (Figure 4F). To validate these findings, we performed real-time RT-PCR and western blot in FAH-silenced or FAH-overexpressing A375 cells. Consistently, results showed that the expression of FH, ME1, PC, PDHB, SLC25A1, SLC25A10, and SLC25A11 was largely decreased after FAH silencing (Figure 4G–4H) and slightly increased after FAH overexpression (Supplementary Figure 4B–4C). These results suggested that FAH activity effectively provides intermediates for the TCA cycle.

**Figure 5:**
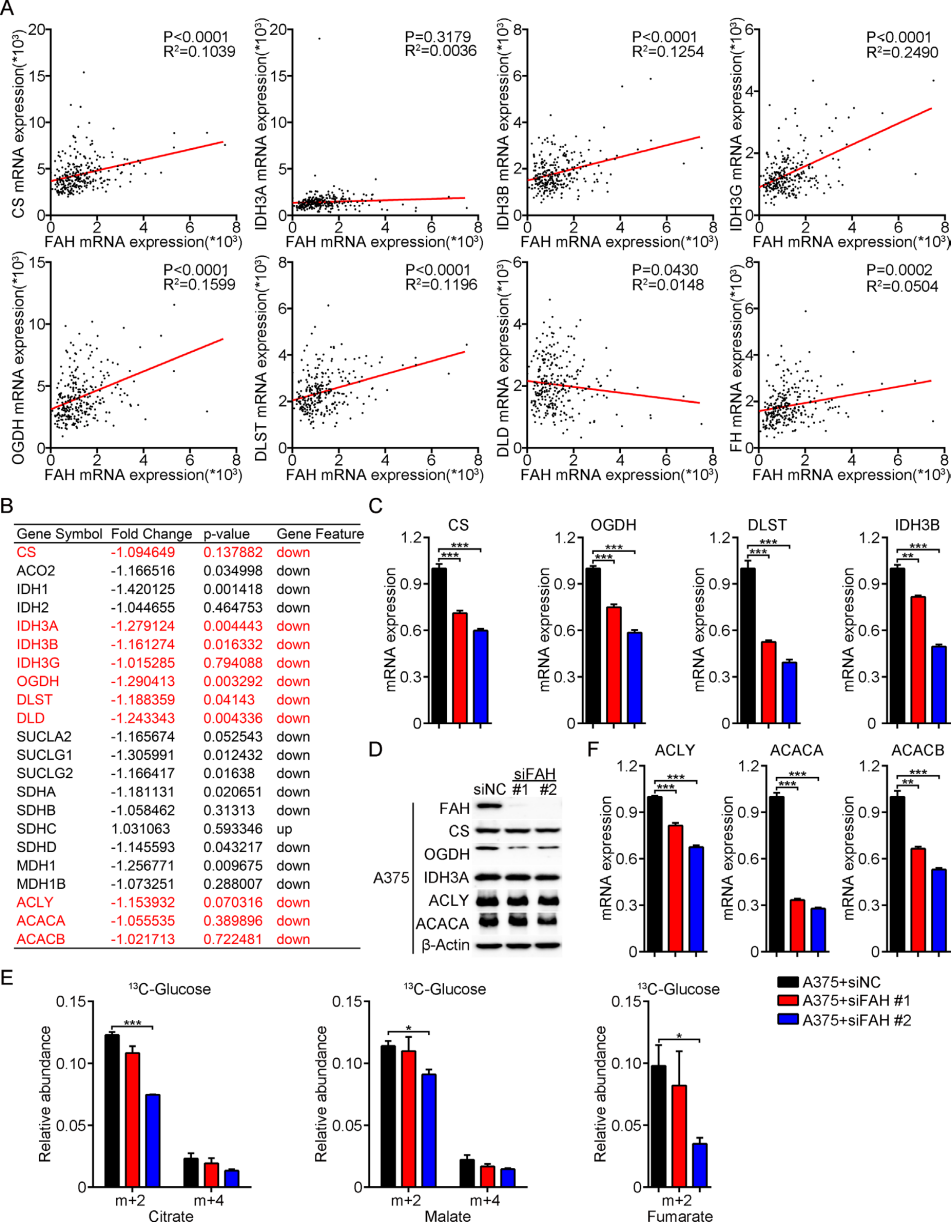
FAH knockdown reduces flux through the TCA cycle and inhibits fatty acids synthesis (**A**) FAH, CS, IDH3A, IDH3B, IDH3G, OGDH, DLST, DLD, and FH mRNA data from clinical samples of melanoma (TCGA database). (**B**) Expression profiling of ACLY, ACACA, ACACB and TCA cycle enzymes’ mRNAs in FAH-silenced A375 cells. (**C**) Quantification of CS, OGDH, DLST, and IDH3B mRNAs by real-time RT-PCR in siFAH-transfected A375 cells. (**D**) Western blot analyses of FAH, CS, OGDH, IDH3A, ACLY, and ACACA expression in siFAH-transfected A375 cells. (**E**) Analysis of the relative abundance of ^13^C-labeled citrate, malate, and fumarate in A375 cells transfected with siFAHs. Cells were cultured with ^13^C-D-glucose for 24 hours and analyzed by LC-MS. (**F**) Quantification of ACLY, ACACA and ACACB mRNAs by real-time RT-PCR in siFAH-transfected A375 cells. Results are expressed as mean ± SEM of samples assessed in triplicates. Representative data from three separate experiments are shown. ^*^p < 0.05; ^**^p < 0.01; ^***^p < 0.001 (Student’s *t-test*).

We next accessed cutaneous melanoma data from TCGA and asked whether FAH can accelerate metabolic flux through the TCA cycle by analyzing, at the mRNA level, the correlation between FAH and the enzymes involved in the TCA cycle. Interestingly, rate-limiting enzymes of the TCA cycle, including citrate synthase (CS), isocitrate dehydrogenase (IDH3A, IDH3B, and IDH3G) and the a-ketoglutarate dehydrogenase complex (OGDH, DLST, and DLD), were all found to be positively associated with FAH (Figure 5A). Moreover, other TCA cycle enzymes, such as malate dehydrogenase (MDH), aconitase (ACO2), succinyl-CoA synthetase (SUCLG1 and SUCLG2), succinate dehydrogenase (SDHA, SDHB, SDHC, and SDHD) and FH, also displayed a positive correlation with FAH (Figure 5A and Supplementary Figure 5A). These results were followed by analyses of mRNA expression profiling in FAH-silenced A375 cells, which showed that the expression of the TCA cycle enzymes mentioned above was indeed downregulated after FAH knockdown (Figure 5B). We next performed real-time RT-PCR and western blot in FAH-silenced or overexpressing A375 cells. As expected, a clear decrease in the expression of rate-limiting enzymes of the TCA cycle was observed after FAH knockdown (Figure 5C–5D) while a slight increase occurred instead in FAH-overexpressing cells (Supplementary Figure 5B-–5C).

To further validate the contribution of FAH to metabolic flow through the TCA cycle, we performed ^13^C tracing by Liquid Chromatography Q-Exactive Mass Spectrometry (LC-QE-MS) in A375 cells using ^13^C-D-glucose. Through this approach, TCA cycle intermediates would obtain after each cycle two ^13^C atoms from acetyl coenzyme A (acetyl-CoA), derived from glycolysis of ^13^C-D-glucose. Hence the relative abundance of intermediates containing two (m+2), four (m+4), or six (m+6) ^13^C atoms represents the metabolic flow rate of the TCA cycle. Results showed that the relative abundance of citrate, malate, or fumarate containing two (m+2) or four (m+4) ^13^C atoms was decreased after FAH knockdown (Figure 5E) and slightly increased after FAH overexpression (Supplementary Figure 5D), indicating deceleration and acceleration, respectively, of the TCA cycle. This suggests that FAH effectively increases TCA cycle activity. Together, these results indicate that FAH stimulates energy metabolism and biosynthesis, affecting multiple biological behaviors in melanoma.

Citrate is an essential intermediate in the TCA cycle that is transported from the mitochondria to the cytoplasm by SLC25A1 [22] to be broken down into acetyl-CoA by the enzyme ATP-citrate lyase (ACLY). Acetyl-CoA, in turn, is converted into malonyl-CoA by acetyl-CoA carboxylases (ACACA and ACACB), which is the rate-limiting step in fatty acid biosynthesis. Since the above studies have demonstrated that FAH effectively promotes anaplerotic reactions, we speculated that FAH activity may also lead to increased citrate levels in mitochondria and subsequently influence fatty acids synthesis. This hypothesis was tested by analyzing mRNA expression profiling data in FAH-silenced A375 cells. Results showed that the expression of ACLY, ACACA, and ACACB were decreased after FAH knockdown (Figure 5B). These findings were corroborated by real-time RT-PCR and western blot, (Supplementary Figure 5D and 5F) which showed increased ACLY, ACACA, and ACACB levels after FAH overexpression (Supplementary Figure 5C and 5E). These data indicated that FAH promotes fatty acids synthesis from citrate in melanoma cells.

### Enhanced FAH expression stimulates glycolysis, the pentose phosphate pathway, and nucleotide synthesis in melanoma cells

**Figure 6:**
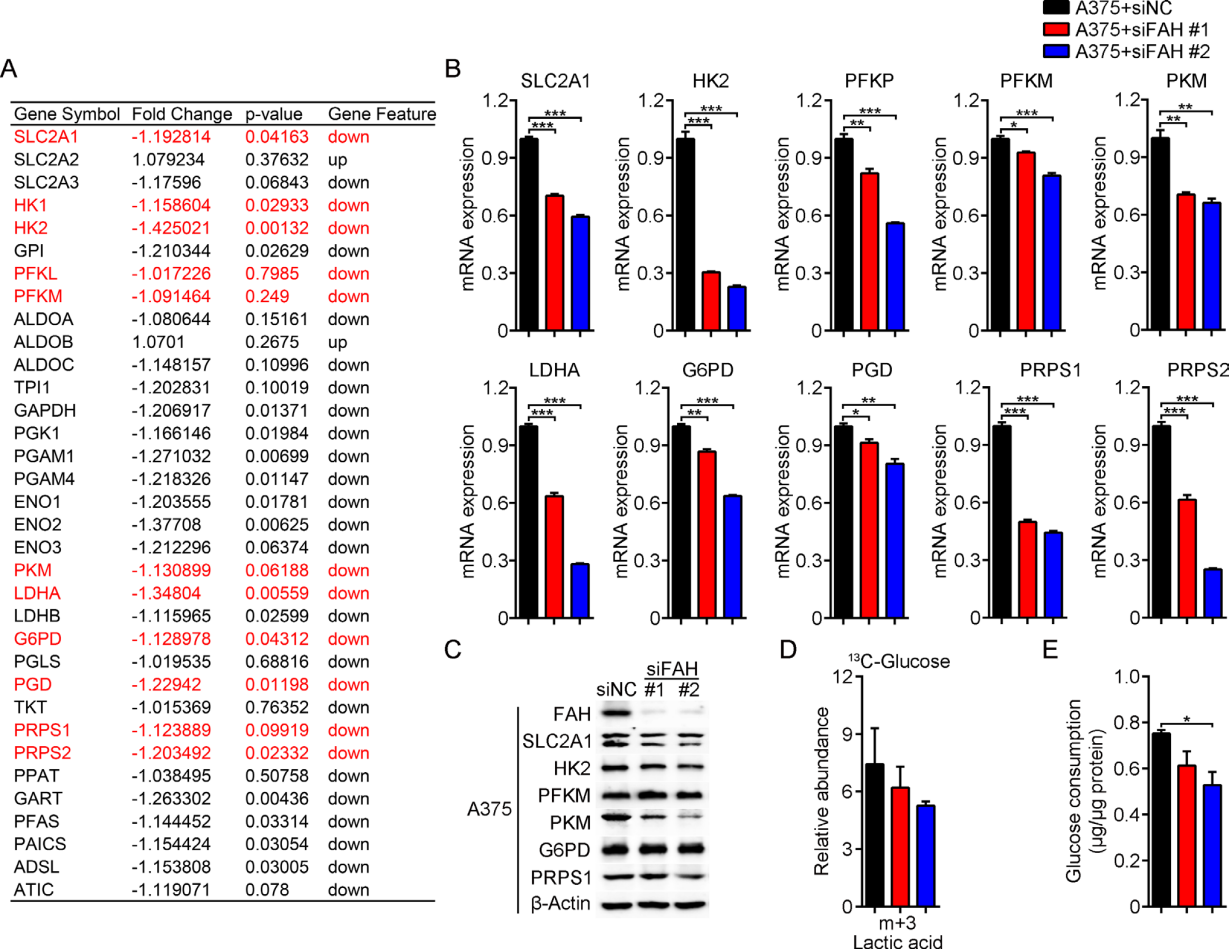
FAH knockdown inhibits glycolysis, pentose phosphate pathway activity, and nucleotide synthesis in melanoma cells (**A**) Expression profiling of glycolytic, PPP, and nucleotide biosynthetic enzymes’ mRNAs in FAH-silenced A375 cells. (**B**) Quantification of SLC2A1, HK2, PFKP, PFKM, PKM, LDHA, G6PD, PGD, PRPS1, and PRPS2 mRNAs by real-time RT-PCR in siFAH-transfected A375 cells. (**C**) Western blot analysis of FAH, SLC2A1, HK2, PFKM, PKM, G6PD, and PRPS1 in siFAH-transfected A375 cells. (**D**) Analysis of the relative abundance of ^13^C-labeled lactate in siFAH-transfected A375 cells. Cells were cultured with ^13^C-D-glucose for 24 hours and analyzed by LC-MS. (**E**) Determination of glucose consumption rate in siFAH-transfected A375 cells. Data are expressed as mean ± SEM; *n* = 3 independent experiments. ^*^p < 0.05; ^**^p < 0.01; ^***^p < 0.001 (Student’s *t*-test).

We next asked if in addition to the TCA cycle, FAH activity may affect other glucose-utilizing metabolic pathways, such as glycolysis and the pentose phosphate pathway (PPP), an important branch of glycolysis that provides the nucleotide precursor ribose 5-phosphate and is a major source of cytosolic NADPH. To address this question, we further analyzed mRNA expression profiling data in FAH-silenced A375 cells. Results showed that levels of the glucose transporters (SLC2A family) and related enzymes involved in glycolysis, the PPP pathway, and nucleotide synthesis, such as the rate-limiting enzymes hexokinase (HK), phosphofructokinase (PFKL, PFKM, and PFKP), pyruvate kinase (PKM), lactate dehydrogenase (LDH), glucose-6-phosphate dehydrogenase (G6PD) and phosphoribosyl pyrophosphate synthetase (PRPS1 and PRPS2), were mostly decreased after FAH knockdown (Figure 6A). In parallel, real-time RT-PCR and western blot analyses confirmed a decrease in the expression of SLC2A1 and the above mentioned enzymes after FAH knockdown (Figure 6B–6C), whereas an increase, in contrast, was observed in A375 cells after FAH overexpression (Supplementary Figure 6A–6C).

Next, ^13^C tracing experiments were done in A375 cells to functionally validate these results. Here, glycolysis intermediates should incorporate three or six ^13^C atoms from ^13^C-D-glucose, hence the relative abundance of lactic acid containing three (m+3) ^13^C atoms represents glycolytic flow rate. In line with mRNA and protein expression results, ^13^C-labeled (m+3) lactate was decreased after FAH knockdown (Figure 6D) and slightly increased after FAH overexpression (Supplementary Figure 6D); these results indicated, respectively, reduced and increased glycolytic rates. In addition, we evaluated glucose consumption rate using enzymatic methods. Results showed that the glucose consumption rate of A375 cells was decreased after FAH knockdown (Figure 6E) and slightly increased after FAH overexpression (Supplementary Figure 6E).

These results demonstrated that FAH effectively stimulates glycolysis, the PPP pathway, and nucleotide synthesis, and increases glucose consumption in melanoma cells.

### CDC5L drives FAH expression in melanoma

**Figure 7:**
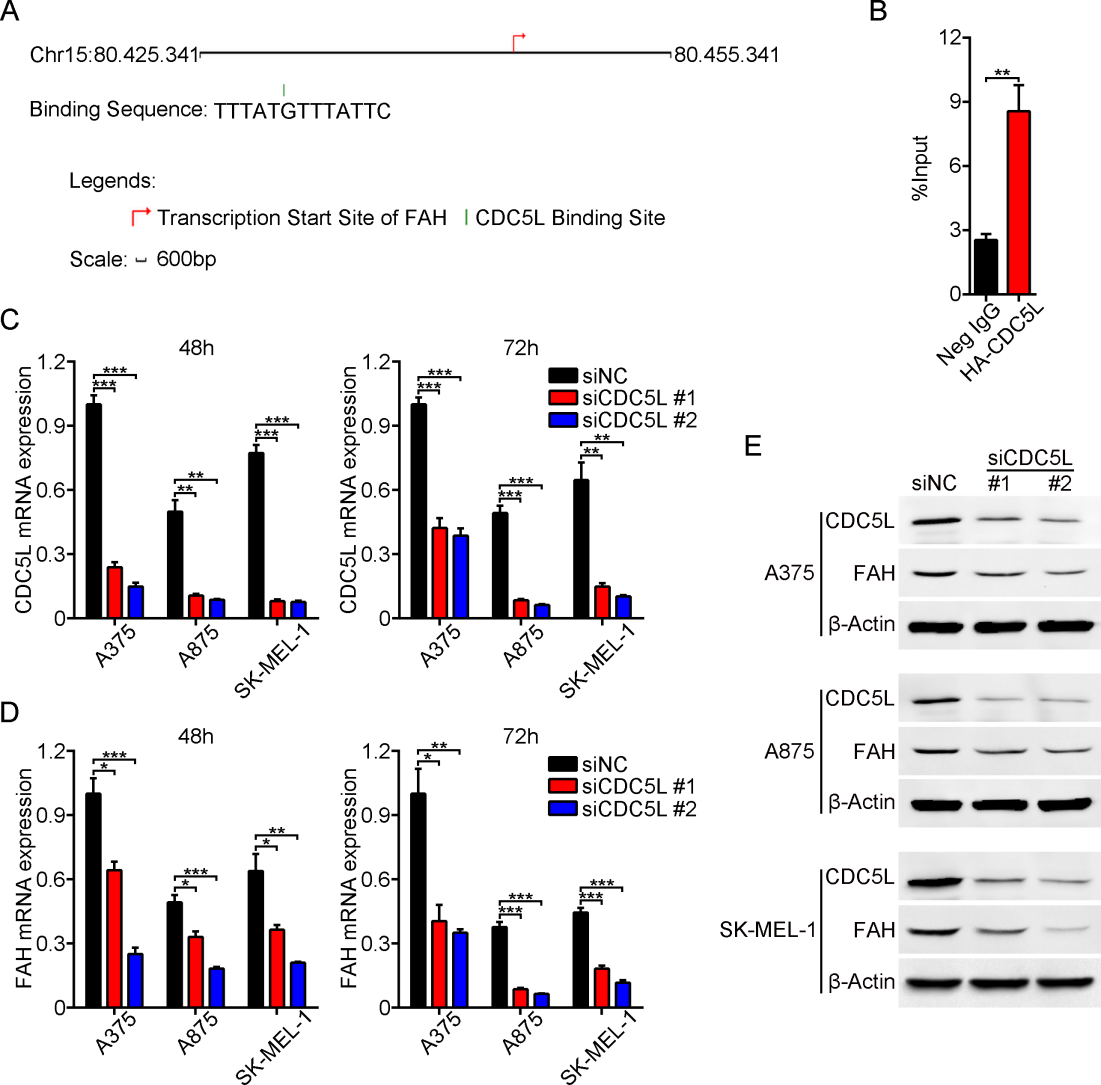
CDC5L drives FAH expression in melanoma cells (**A**) CDC5L binding sequence and location on the *FAH* gene, determined using the SABiosciences database. (**B**) ChIP-qPCR assay. DNA fragments interacting with CDC5L protein were pulled down by an anti-HA antibody and amplified by real-time PCR. Total chromatin was used as input. Normal human IgG served as negative control. (**C–E**) Silencing efficiency of two CDC5L siRNAs assessed by real-time RT-PCR (C) and western blot (E) at 48 and 72 h, respectively. These samples were then used to detect FAH expression using real-time RT-PCR (D) and western blot (E). Data are expressed as the mean ± SEM of three independent experiments. ^*^p < 0.05; ^**^p < 0.01; ^***^p < 0.001 (Student’s *t-test*).

Mammalian cell division cycle 5-like protein (CDC5L), a functional homolog of the Schizosaccharomyces pombe *cdc5* gene, has been shown to act as a positive regulator of G2/M progression and is a basic component of the non-snRNA spliceosome [27, 28]. In addition, CDC5L shares significant homology with the transcription factor c-Myb, indicating a potential role in regulating gene transcription [29, 30]. Upon searching the SABiosciences proprietary database (using the SABiosciences Text Mining Application and the UCSC Genome Browser) we found that CDC5L is one of the major predicted transcriptional factors for the *FAH* gene. The binding site of CDC5L on the *FAH* gene is located at chr15: 80430676-80430688 and the binding sequence is as follows: TTTATGTTTATTC (Figure 7A). To test the hypothesis that CDC5L is an important transcription factor for the *FAH* gene [31], chromatin immunoprecipitation (ChIP) and real-time PCR analysis were performed in melanoma cells. Because specific immunoprecipitation human CDC5L antibodies are not available, we constructed recombinant plasmids containing hemagglutinin- (HA) tagged CDC5L (pCMV-C-HA-CDC5L-CDS) and validated them by direct DNA sequencing (data not shown). Then we transfected this vector into A375 cells and conducted ChIP-qPCR using primers for the predicted binding sequence of the *FAH* gene (Supplementary Table 3). Clear amplification of a 120-bp DNA region including the predicted binding site was obtained from anti-HA precipitates, whereas anti-IgG antibody precipitates yielded negligible amplification of this DNA region (Figure 7B). These data indicated that CDC5L protein specifically binds to the *FAH* gene *in vitro*. To further confirm that CDC5L increases FAH expression, we knocked down the *Cdc5l* gene in melanoma cells using two siRNAs (Figure 7C and 7E), and analyzed FAH expression by real-time RT-PCR and western blot. Results showed that the expression of FAH was significantly decreased at both the mRNA (Figure 7D) and protein (Figure 7E) levels at different time points, suggesting that CDC5L effectively drives the expression of the *FAH* gene.

## DISCUSSION

Anaplerotic reactions are essential for replenishing TCA cycle intermediates; they maintain the homeostasis of multiple metabolic pathways and are crucial for the survival and proliferation of tumor cells. The production of oxaloacetate from pyruvate by PC is considered the most physiologically important event within these pathways. Although multiple anaplerotic reactions have been identified in tumor cells, many of the enzymes involved remain to be identified.

In this study, we found that FAH is highly expressed in clinical specimens of melanoma, where it correlates with shortened survival times and may thus serve as an independent prognostic biomarker. Subsequently, our in vitro studies using three human melanoma cell lines revealed that FAH regulates important biological processes such as proliferation, migration, and apoptosis. Furthermore, we show that disrupting FAH in melanoma cells effectively prolongs survival in tumor-bearing mice. We attribute the pro-tumoral actions of FAH to its ability to promote anaplerotic reactions that underlie metabolic reprogramming in melanoma cells.

Little is known about the global metabolic impact of FAH on tumor cells. FAH catalyzes the conversion of 4-fumarylacetoacetate to acetoacetate and fumarate from L-phenylalanine and tyrosine catabolism. Through FAH knockdown and overexpression experiments combined with real time RT-PCR, western blot, and ^13^C metabolic flux analyses, the present study reveals that a remarkable number of enzymes within the glycolytic, TCA cycle, PPP, and nucleotide biosynthetic pathways are affected by fluctuations in cellular FAH levels in melanoma cells.

Based on sequence interaction evidence from genome data analyses, our ChIP-qPCR analysis confirmed that CDC5L binds to the *FAH* gene and drives FAH expression in A375 cells. Accordingly, disrupting CDC5L using siRNA significantly downregulated FAH levels. A transcription factor function for CDC5L has been previously suggested, but not clearly proved, based on sequence homology with c-Myb [29, 30]. Interestingly, since the binding site of CDC5L on the *FAH* gene is at a relatively distant position upstream of the transcription initiation point, i.e. at an unusual region of the gene’s promoter, we speculate that CDC5L might not bind to FAH’s promoter, but to other cis-regulatory elements (CREs) such as enhancers or silencers [32–34]. Thus, although the present data indicate that CDC5L is a putative transcription factor for FAH, detailed confirmatory experiments are warranted. Our next studies will also address the potential role of FAH in the regulation of hypoxia-inducible factor (HIF) target genes, as the elevated level of fumarate derived from increased FAH expression are likely to inhibit prolyl hydroxylase (PHD), preventing HIF degradation [35–38]. Stabilized HIF-1α is translocated to the nucleus and activates transcription of target genes including genes of glycolysis, glucose uptake and other metabolic pathways [39–41].

In summary, consistent with clinical evidence linking FAH overexpression with shortened melanoma patient survival, our study revealed that FAH plays a critical role in melanoma cell growth and survival by stimulating anaplerotic reactions that lead to increased energy metabolism and enhanced biosynthesis of fatty acids and nucleotides. The present study offers novel insights into melanoma’s metabolic reprogramming, and suggests that FAH is both a useful independent prognostic biomarker and a relevant target for melanoma therapy.

## MATERIALS AND METHODS

### Cell lines and animals

Human melanoma A375 cells were a kind gift of the Department of Oncology, Union Hospital (Wuhan, China). A875 and SK-MEL-1 cells were purchased from the Cell Resource Center, Institute of Basic Medical Sciences, CAMS/PUMC (Beijing, China). All cells were checked for mycoplasma and interspecies cross-contamination, and authenticated by short tandem repeat (STR) profiling and isoenzyme analyses at the Cell Resource Center and at the China Center for Type Culture Collection (Wuhan, China) before the study started, and occasionally during the course of this research. A375, A875, and SK-MEL-1 cells were cultured in Dulbecco’s Modified Eagle Medium (DMEM, Gibco, Grand Island, NY) supplemented with 10% fetal bovine serum (Gibco). All cell lines were maintained in culture for a maximum of 20 passages (two months). Female BALB/c-nude mice (5 to 7 weeks old) were purchased from HFK Bioscience (Beijing, China). Animal studies were approved by the Animal Care and Use Committee of Tongji Medical College (Wuhan, China).

### RNA interference

A375 and A875 cells stably expressing short hairpin RNAs (shRNAs) against FAH and GFP were generated through lentiviral infection and puromycin selection. The shRNA-expressing virus was constructed by GeneChem (Shanghai, China), and direct sequence analysis was used to verify the inserted sequences. Melanoma cells were stably transduced with viral supernatant (MOI = 10, diluted to 10^7^ TU/ml with 10% FBS/DMEM; 8 μg/ml polybrene) and fresh 10% FBS/DMEM was added after 12 h. Thereafter, selection with 2 μg/ml of puromycin was initiated at 48 h and allowed to continue for 3 to 5 days. Stably transduced cells were maintained in 10% FBS/DMEM with 1 μg/ml puromycin. Silencing efficiency was verified by GFP detection, western blot, and real-time RT-PCR.

For FAH and CDC5L knockdown, siRNAs against these two genes and negative control siRNAs were purchased from RiboBio (Guangzhou, China). The corresponding sequences are shown in Supplementary Table 3. Cells were transfected with siRNAs using Lipofectamine 3000 (Invitrogen, Carlsbad, CA, USA) at a final concentration of 50 nmol/L. Silencing efficiency was verified by western blot and real-time RT-PCR.

### Microarray assay

Total RNA from A375 cells transfected with FAH-targeted siRNA (siFAH) or negative control siRNA was extracted using the miRNeasy Mini Kit (Qiagen, Hilden, Germany) and further purified with an RNase-Free DNase Set. RNA quality was assessed by agarose gel electrophoresis. Subsequently, biotinylated cDNA was prepared according to the standard Affymetrix (Santa Clara, CA, USA) protocol from 250 ng of total RNA using a GeneChip^®^ WT PLUS Reagent Kit. Following labeling, 5.5 μg of cDNA was hybridized for 16 h at 45°C on a GeneChip Human Gene 1.0 ST Array in a GeneChip® Hybridization Oven 645. GeneChips were washed and stained in an Affymetrix Fluidics Station 450. We assayed three biological replicates for each sample. All arrays were then scanned using a GeneChip® Scanner 3000 (Affymetrix). Data were analyzed with the Robust Multichip Analysis (RMA) algorithm using Affymetrix default analysis settings and global scaling as the normalization method.

### Bioinformatic analysis

DEGs were identified based on SAM (Significance Analysis of Microarrays). Afterwards, we selected DEGs based on ≥ 1.2-fold-change in either direction, and DEGs for which *p* < 0.05 were considered downregulated or upregulated.

Gene Ontology (GO) analysis was applied for DEG functional analysis. Statistical significance (defined as *p* < 0.05) was assessed using Fisher’s exact test and multiple comparison tests, with a false discovery rate (FDR) < 0.05 as a cut-off. We calculated the enrichment (Re) score to determine the enrichment level per GO term using the following formula: Re = (n_f_/n)/(N_f_/N), where n_f_ is the number of target genes within a particular category, n is the total number of genes within the same category, N_f_ is the number of target genes in the entire microarray, and N is the total number of genes in the microarray. Kyoto Encyclopedia of Genes and Genomes (KEGG) pathway analysis was used to identify significant pathways related to the DEGs. Again, we used Fisher’s exact test and multiple comparison test to select significant pathways with *p* < 0.05. Re was calculated using the equation above. GO and KEGG pathway analyses were conducted using the online analysis tool Gene Cloud of Biomedical information (GCBI; https://www.gcbi.com.cn). The microarray data presented in this study have been deposited in the NCBI Gene Expression Omnibus and are accessible through GEO Series accession number GSE86870 (http://www.ncbi.nlm.nih.gov/geo/query/acc.cgi?acc=GSE86870).

Human melanoma tissue samples’ mRNA values were obtained from the Oncomine database. Survival data and related gene expression values from melanoma patients were obtained from The Cancer Genome Atlas (TCGA) database. The binding position and sequence of CDC5L in the *FAH* gene were obtained from the SABiosciences proprietary database.

### Plasmid constructs and transfection

FAH overexpression vectors were constructed by Goodbio Company (Wuhan, China) by inserting human FAH cDNA into pcDNA3.1 plasmids. The human CDC5L (NM_001253.3) CDS without the stop codon was amplified, double-digested with SmaI and XbaI, and then cloned into the pCMV-C-HA vector (Beyotime, Shanghai, China) to construct the pCMV-C-HA-CDC5L-CDS vector. Lipofectamine 3000 (Invitrogen) was used to transfect the plasmids into melanoma cells. All constructs were subjected to direct sequence analysis to verify the identities of the inserted sequences.

### *In vitro* migration assay

Complete medium (DMEM containing 10% FBS; 600 μl) was added to the lower chamber of 24-well transwell plates (Corning, NY, USA). Then, 5 × 10^4^ melanoma cells resuspended in 100 μl of serum-free DMEM were seeded into the upper chambers in triplicate and incubated at 37°C. A375 cells were incubated for 24 h, whereas A875 cells were incubated for 48 h because of their lower migration capacity. After being washed with phosphate-buffered saline (PBS), the cells were fixed using 4% neutral paraformaldehyde for 30 min and stained with 0.1% crystal violet for 20 min. Tumor cells migrating into the lower surface of the membranes were photographed at 100x magnification. Cells in ten random fields were counted to quantify migration.

### Cell cycle analysis

Cells were alternatively transfected with two siFAH or FAH-overexpressing plasmids for 60 h, trypsinized, washed with PBS, and fixed with cold (–20°C) 70% ethanol. After 20 h, cells were labeled using a Cell Cycle Detection Kit (KeyGen Biotech, Nanjing, China) according to the manufacturer’s instructions. Cell DNA contents were measured on an Accuri C6 flow cytometer (BD Biosciences, Ann Arbor, MI, USA). The percentages of cells in G1, S, and G2 phases were determined using FlowJo 7.6 software.

### Apoptosis analysis

Cells were alternatively transfected with two siFAH or FAH-overexpressing plasmids for 60 h and labeled using an Annexin V-FITC Apoptosis Detection Kit (KeyGen Biotech) according to the manufacturer’s protocol. Apoptosis levels were analyzed on an Accuri C6 flow cytometer.

### Semi-quantitative PCR and real-time RT-PCR

Cells were lysed in TRIzol reagent (Invitrogen), and total RNA was extracted following the manufacturer’s instructions. Real-time PCR analyses were performed with SYBR Green PCR master mix (Toyobo, Osaka, Japan) using a Bio-Rad CFX Connect™ Real-Time PCR Detection System (Bio-Rad, Mississauga, ON, Canada). Comparative quantitative mRNA levels were normalized to the housekeeping gene β-actin. Primer sequences are shown in Supplementary Table 3.

### Cellular fumarate detection

Total cellular fumarate levels were determined using the Fumarate Detection Assay Kit (Sigma-Aldrich, St. Louis, MO, USA) following the manufacturer’s protocol. Briefly, cells were homogenized in Fumarate Assay Buffer and then centrifuged to remove insoluble material. Then we set up the Master Reaction Mix which including Fumarate Developer, Enzyme Mix, and Assay Buffer. The samples and the Master Reaction Mix were mixed, incubated at 37°C for 60 min, after which absorbance was measured at 450 nm.

### Glucose consumption assay

Melanoma cells were alternatively transfected with two siFAH or FAH-overexpressing plasmids for 24 h. After complete medium exchange, a cell-free control group was set. Following a 24 h incubation, the supernatants were collected and glucose concentration was measured using a Glucose Assay Kit (Sigma) according to the manufacturer’s instructions. Cells were lysed for protein quantification using a BCA Protein Assay Kit (Thermo Scientific, Rockford, IL, USA). Glucose consumption rate was determined as the glucose concentration in the supernatants minus that of the cell-free control group, and normalized to total protein levels.

### Tissue microarray and immunohistochemistry

A human melanoma tissue microarray was purchased from Alenabio Biotechnology Co. (Xi’an, China). Immunohistochemistry using an anti-human FAH antibody (Abcam, Cambridge, MA, USA) was conducted by Goodbio Co. (Wuhan, China). IOD values of microarray samples were determined with IPP 6.0 software. Detailed information on the patient samples included in the microarray and corresponding IOD values is provided in Supplementary Table 1.

### Western blot analysis

Cytosolic and mitochondrial fractions were isolated from melanoma cells using the Cell Mitochondria Isolation Kit (Beyotime, Shanghai, China). Western blot was performed as described previously [42]. Proteins were extracted and analyzed using antibodies targeting the following human proteins: FAH (Abcam), COX-IV (Beyotime), FH, ME1, PC, PDHB, CS, OGDH, IDH3A, ACACA, ACLY, HK2, PFKM, PKM, SLC2A1, G6PD, PRPS1, CDC5L (Proteintech, Wuhan, China), N-Cadherin, E-Cadherin, TCF8/ZEB1 (Cell Signaling Technology, Beverly, MA, USA), and β-actin (Abcam) at the recommended dilutions.

### Two-photon confocal microscopy

A375 cells were stained with anti-human FAH (Abcam) and anti-human COX-IV (Beyotime) antibodies according to the manufacturer’s instructions and visualized by two-photon confocal microscopy (Leica, Wetzlar, Germany).

### ^13^C tracing by Liquid Chromatography Q-Exactive Mass Spectrometry (LC-QE-MS)

For ^13^C tracing experiments, A375 cells were alternatively transfected with two siFAHs or FAH-overexpressing plasmids for 48 h. After washing, cells were cultured with D-glucose (U-^13^C6, 99%; Cambridge Isotope Laboratories, Inc., Andover, MA, USA) for 24 h. Cells were trypsinized, washed twice with PBS and lysed in extraction solvent (80% methanol/water) for 30 min at –80°C. After centrifugation at 13,000 g (10 min at 4°C), supernatant extracts were analyzed using LC-QE-MS. Briefly, liquid chromatography was performed using a HPLC system (Ultimate 3000 UHPLC) (Thermo Fisher Scientific, San Jose, CA, USA) equipped with an XBridge amide column (100 × 2.1 mm i.d., 3.5 μm; Waters). The column temperature was maintained at 10 °C. The mobile phase A was 20 mM ammonium acetate and 15 mM ammonium hydroxide in water with 3% acetonitrile, pH 9.0, and the mobile phase B was acetonitrile. The linear gradient was as follows: 0 min, 85% B, 1.5 min, 85% B, 5.5 min, 30% B, 8 min, 30% B, 10 min, 85% B, and 12 min, 85% B. The flow rate was 0.2 mL/min. Sample volumes of 5 μl were injected for LC-MS analysis on a Q-Exactive mass spectrometer (Thermo Fisher Scientific) equipped with a HESI probe; the relevant parameters were: heater temperature, 120 °C; sheath gas, 30; auxiliary gas, 10; sweep gas, 3; and spray voltage, 2.5 kV for the negative mode. A full scan ranging from 80 to 350 (m/z) was used. Resolution was set at 70,000. Data were quantified by integrating the area under the curve of each compound using Xcalibur Qual Browser (Thermo Fisher). Each metabolite’s accurate mass ion and subsequent isotopic ions were extracted (EIC) using a 10 ppm window.

### Chromatin immunoprecipitation (ChIP) and qPCR analysis

As specific anti-human CDC5L antibodies for immunoprecipitation studies are not available, we constructed pCMV-C-HA-CDC5L-CDS plasmids and transfected them into A375 cells using Lipofectamine 3000. ChIP was performed using the EZ-ChIP Kit (Millipore, Billerica, MA, USA). The AVCX130 system (Sonics & Materials, Newtown, CT, USA) was used for cell sonication. Anti-HA (Abcam) and normal anti-human-IgG (Millipore) antibodies were used for immunoprecipitation reactions. The DNA fragments recovered from immunoprecipitated complexes were amplified via real-time PCR (qPCR). The primer sequences are listed in Supplementary Table 3.

### Statistical analysis

Statistical analysis was performed using SPSS 17.0 or GraphPad Prism 6.0 statistical software. All data are presented as mean ± standard error of the mean (SEM). Differences in mRNA expression between groups were calculated using a two-tailed Student’s *t*-test. Survival curves were plotted using the Kaplan-Meier method and compared by the log-rank test. For all tests, statistical significance was set at *p* < 0.05.

## SUPPLEMENTARY MATERIALS FIGURES AND TABLES







## Abbreviations

FAH: fumarylacetoacetate hydrolase
FH: fumarase
acetyl-CoA: acetyl coenzyme A
SLC25A1: solute carrier family 25 (mitochondrial carrier; citrate transporter), member 1
SLC25A10: solute carrier family 25 (mitochondrial carrier; dicarboxylate transporter), member 10
SLC25A11: solute carrier family 25 (mitochondrial carrier; oxoglutarate carrier), member 11
ME: malic enzyme
PC: pyruvate carboxylase
PDHX: pyruvate dehydrogenase complex, component X
PDPR: pyruvate dehydrogenase phosphatase regulatory subunit
PDHB: pyruvate dehydrogenase beta
PDHA1: pyruvate dehydrogenase alpha 1
CS: citrate synthase
ACO2: aconitase 2
IDH: isocitrate dehydrogenase
OGDH: oxoglutarate dehydrogenase
DLST: dihydrolipoamide S-succinyltransferase
DLD: dihydrolipoamide dehydrogenase
SUCL: succinate-CoA ligase
SDH: succinate dehydrogenase complex
MDH: malate dehydrogenase
ACLY: ATP citrate lyase
ACACA: acetyl-CoA carboxylase alpha
ACACB: acetyl-CoA carboxylase beta
SLC2A1: solute carrier family 2 (facilitated glucose transporter), member 1
HK: hexokinase
GPI: glucose-6-phosphate isomerase
PFK: phosphofructokinase
ALDO: aldolase
TPI: triosephosphate isomerase
GAPDH: glyceraldehyde-3-phosphate dehydrogenase
PGK: phosphoglycerate kinase
PGAM: phosphoglycerate mutase
ENO: enolase
PKM: pyruvate kinase
LDH: lactate dehydrogenase
G6PD: glucose-6-phosphate dehydrogenase
PGLS: 6-phosphogluconolactonase
PGD: phosphogluconate dehydrogenase
TKT: transketolase
PRPS: phosphoribosyl pyrophosphate synthetase
PPAT: phosphoribosyl pyrophosphate amidotransferase
GART: phosphoribosylglycinamide formyltransferase, phosphoribosylglycinamide synthetase, phosphoribosylaminoimidazole synthetase
PFAS: phosphoribosylformylglycinamidine synthase
PAICS: phosphoribosylaminoimidazole succinocarboxamide synthetase
ADSL: adenylosuccinate lyase
ATIC: 5-aminoimidazole-4-carboxamide ribonucleotide formyltransferaseIMP cyclohydrolase
DEGs: differentially expressed genes
TCGA: The Cancer Genome Atlas
OS: overall survival
DFS: disease-free survival
GFP: green fluorescent protein
siRNA: small interfering RNA
shRNA: short hairpin RNA
TCA cycle: tricarboxylic acid cycle
real-time RT-PCR: quantitative reverse transcription-PCR
PPP: pentose phosphate pathway
CDC5L: Cell division cycle 5-like protein
CDS: coding DNA sequence
HA: hemagglutinin
CREs: cis-regulatory elements
HIF: hypoxia-inducible factor
PHD: prolyl hydroxylase
STR: short tandem repeat
GO: Gene Ontology
KEGG: Kyoto Encyclopedia of Genes and Genomes
cDNA: complementary DNA
IOD: integrated optical density
