# Supplementary material for: CDC5L drives FAH expression to promote metabolic reprogramming in melanoma
Source: Oncotarget. 2017 Dec 7;8(69):114328–43. doi: 10.18632/oncotarget.23107 (PMC5768407; doi:10.18632/oncotarget.23107)
Supplement: Supplementary file 1 [file oncotarget-08-114328-s001.pdf]

CDC5L drives FAH expression to promote metabolic reprogramming in melanoma

SUPPLEMENTARY MATERIALS

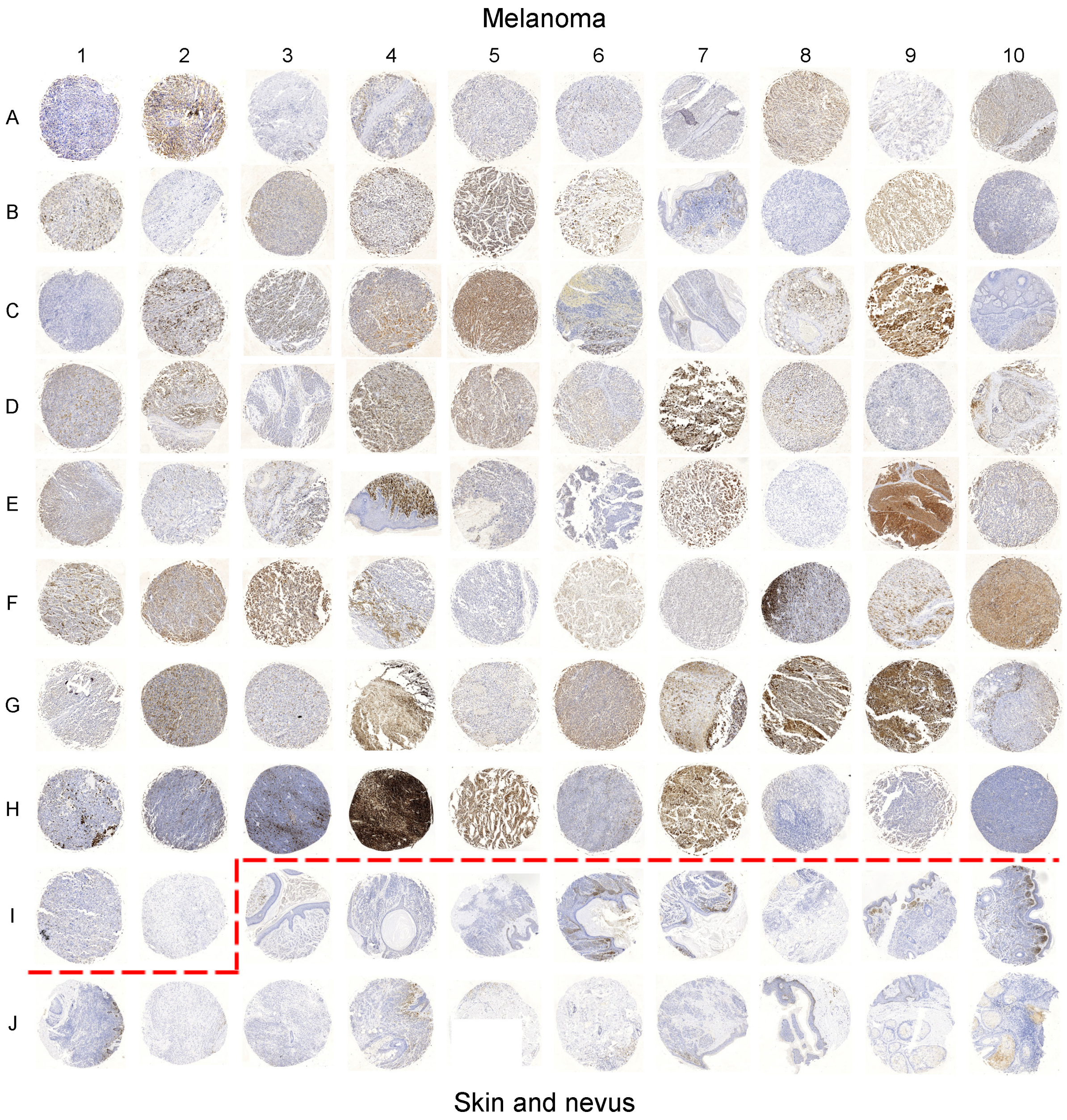

Supplementary Figure 1: Immunohistochemical detection of FAH in a human melanoma tissue microarray.

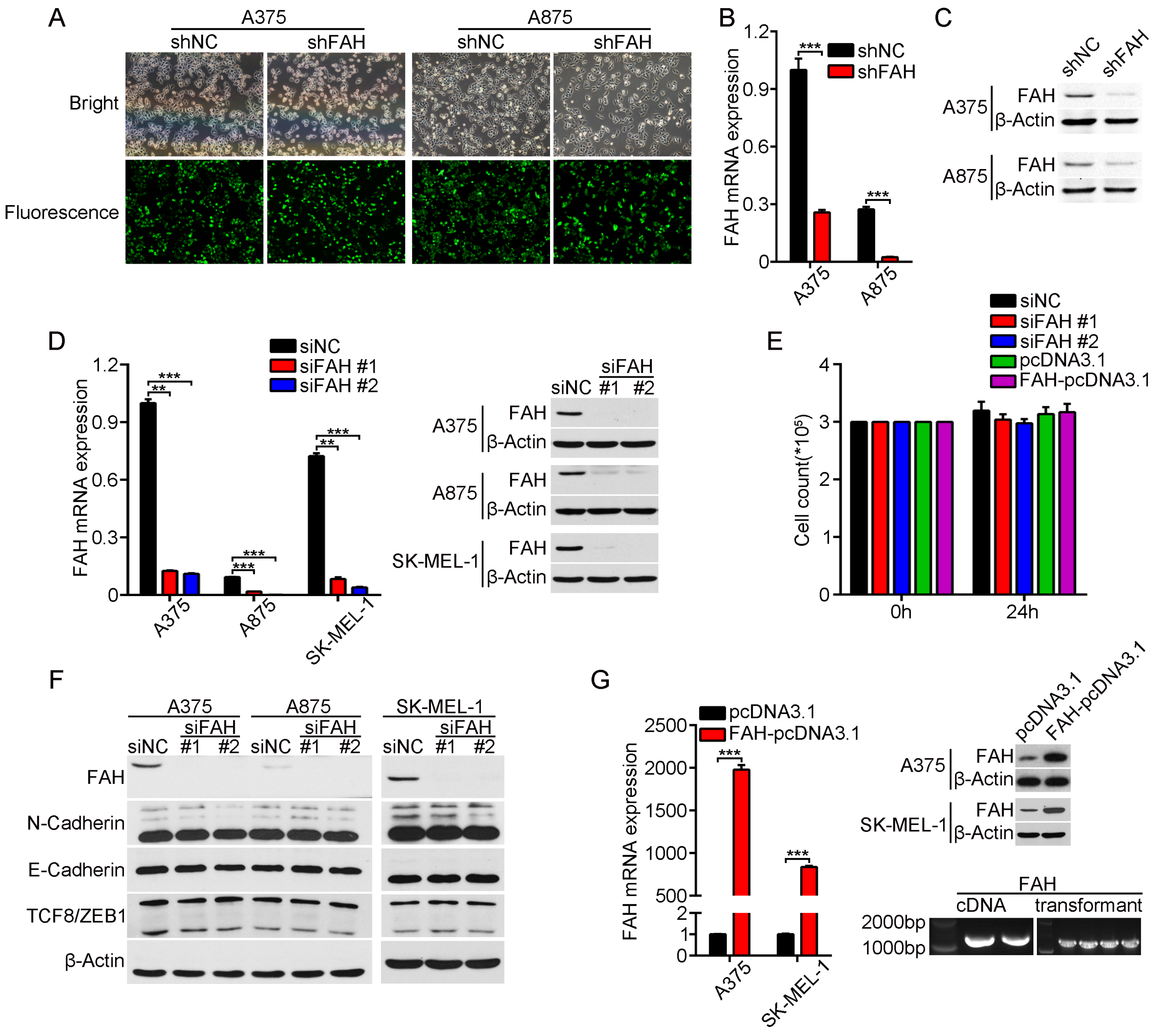

Supplementary Figure 2: Efficiency of FAH-shRNA lentivirus, FAH-siRNAs, and FAH-overexpression plasmids. (**A–C**) Stable FAH knockdown in A375 and A875 cells was achieved by transduction with shFAH or control lentivirus followed by puromycin selection. Transduction efficiency was verified by GFP detection using fluorescence microscopy (A), real-time RT-PCR (B), and western blot (C). (**D**) Melanoma cells were alternatively transfected with two FAH siRNAs or a scrambled siRNA control for 72 h and analyzed using real-time RT-PCR or western blot. (**E**) A375 cells were alternatively transfected with two FAH siRNAs, FAH-overexpression plasmids or control vectors. Then cells (3×10^5) were cultured in serum-free DMEM and cell counts registered 24 h later. (**F**) Melanoma cells were alternatively transfected with two FAH siRNAs or negative control siRNA for 72 h and analyzed for N-cadherin, E-cadherin, and TCF8/ZEB1 using western blot. (**G**) Melanoma cells were transfected with FAH-overexpression or control plasmids for 72 h. Thereafter, cells were lysed for real-time RT-PCR or western blot. FAH cDNA and transformants with pcDNA3.1 plasmids were assessed by agarose gel electrophoresis. Data are expressed as mean ± SEM; $n = 3$ independent experiments; $^{**}p < 0.01$; $^{***}p < 0.001$ (Student's t-test).

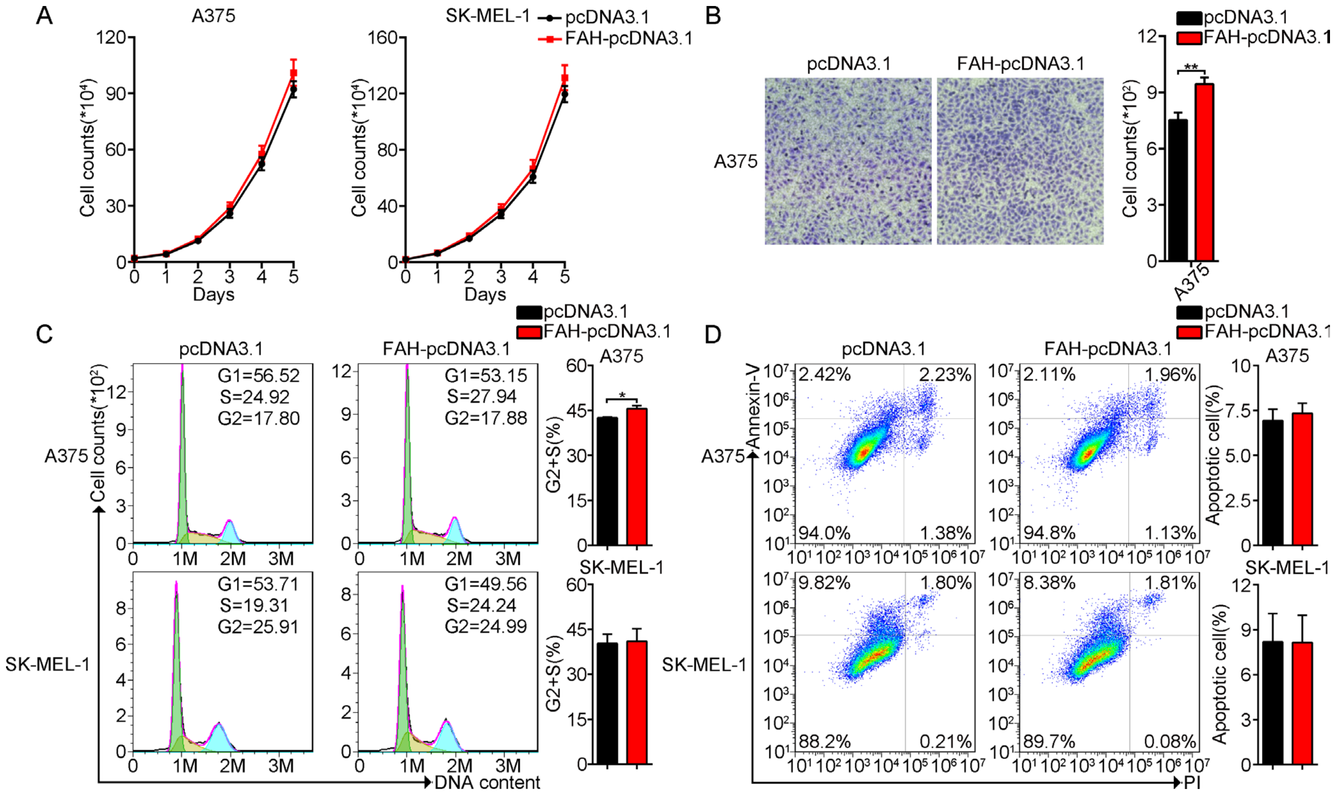

Supplementary Figure 3: Forced overexpression of FAH promotes migration and cell cycle progression in melanoma cells. (**A**) A375 and SK-MEL-1 cells were transfected with FAH-overexpression or control plasmids for 24 h and cultured in 6-well plates (2×10^4 cells/well). Cell numbers were counted at different time points. (**B**) A375 cells were transfected with FAH-overexpression plasmids or control vectors for 72 h before performing transwell assays (5×10^4 cells/well). (**C–D**) Melanoma cells were transfected with FAH-overexpression or control plasmids for 60 h and then cell cycle distribution and apoptosis were analyzed using flow cytometry. Data are expressed as mean ± SEM; $n = 3$ independent experiments; *$p < 0.05$; **$p < 0.01$ (Student's t-test).

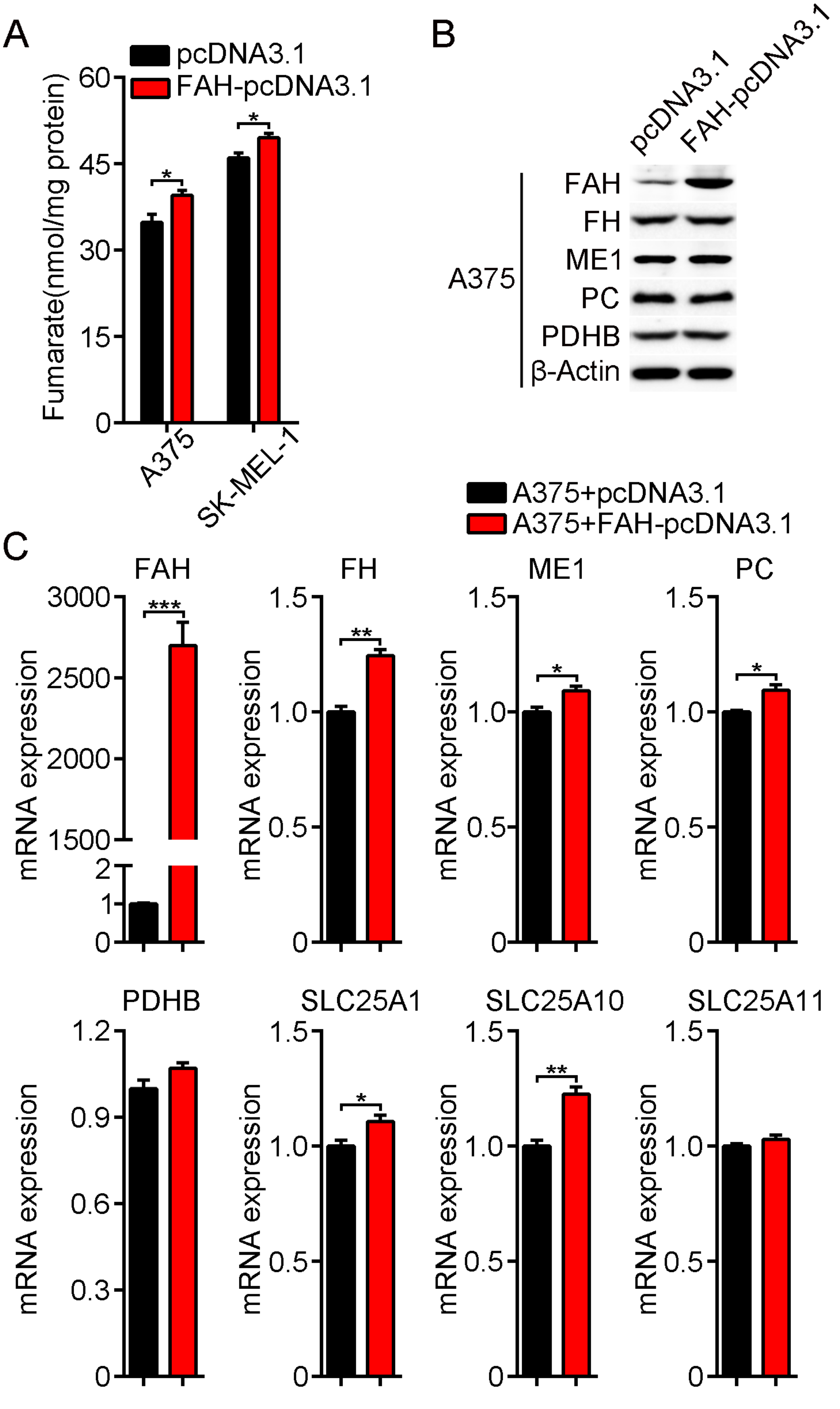

Supplementary Figure 4: Enhanced FAH expression increases TCA cycle activation. A. Melanoma cells were transfected with FAH-overexpression or control vectors for 72 h. Then total cellular fumarate levels were determined using the Fumarate Detection Assay Kit. B. A375 cells were transfected with FAH-overexpression or control plasmids for 72 h and then used for detection of FAH, FH, ME1, PC, and PDHB by western blot. C. A375 cells were transfected with FAH-overexpression or control plasmids for 56 h and then FAH, FH, ME1, PC, PDHB, SLC25A1, SLC25A10, and SLC25A11 mRNAs were quantified using real-time RT-PCR. Data are expressed as mean ± SEM; $n = 3$; $^{*}p < 0.05$; $^{**}p < 0.01$; $^{***}p < 0.001$ (Student's t-test).

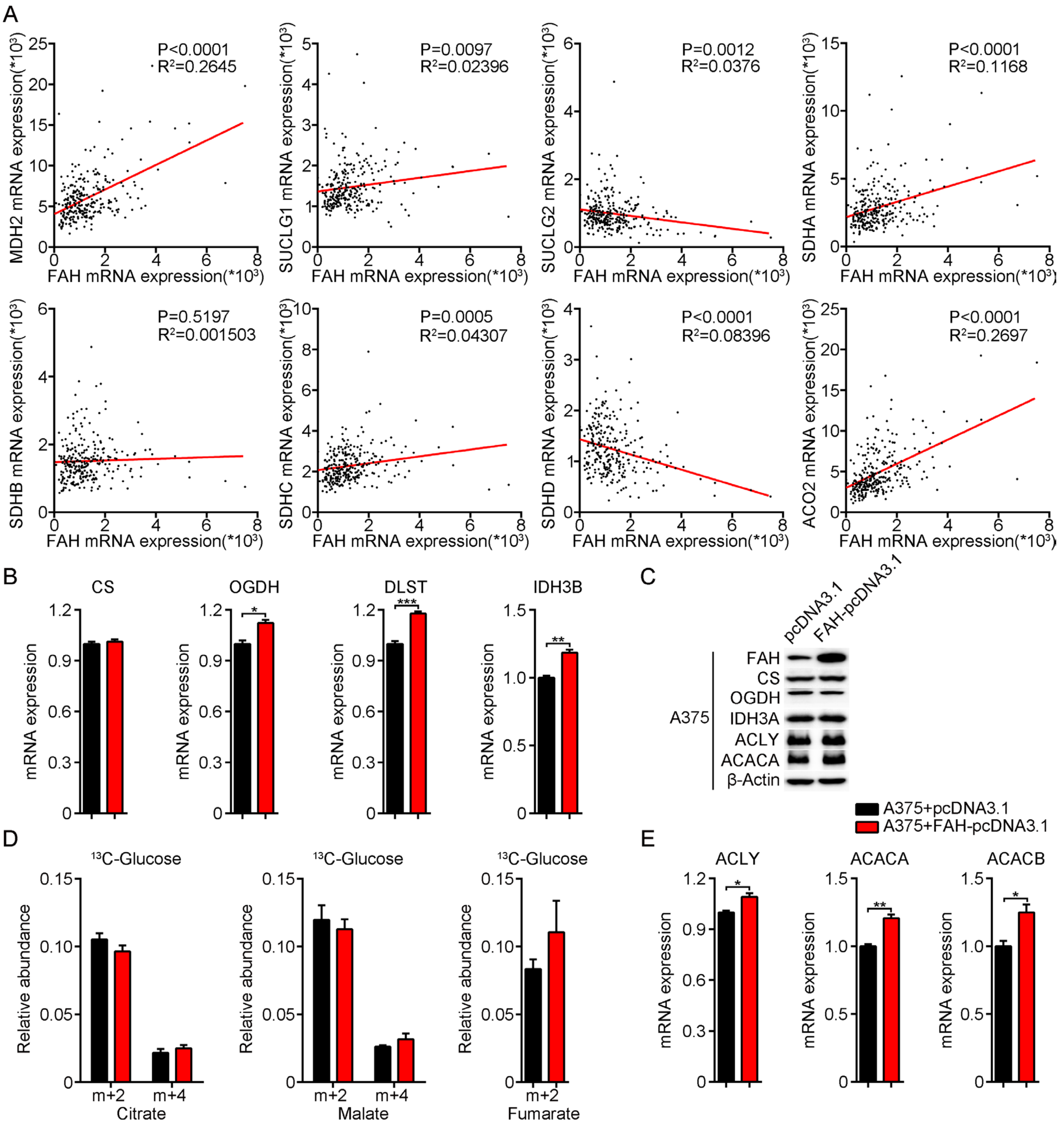

Supplementary Figure 5: Enhanced FAH expression increases TCA cycle fluxes and fatty acids synthesis. (**A**) Quantification of FAH, MDH2, SUCLG1, SUCLG2, SDHA, SDHB, SDHC, SDHD, and ACO2 mRNAs from melanoma clinical specimens (TCGA database). (**B**) A375 cells were transfected with FAH-overexpression or control plasmids for 56 h and CS, OGDH, DLST, and IDH3B mRNAs were quantified using real-time RT-PCR. (**C**) A375 cells were transfected with FAH-overexpression or control plasmids for 72 h and FAH, CS, OGDH, IDH3A, ACLY, and ACACA expression was assessed by western blot. (**D**) Relative abundance of citrate, malate or fumarate containing two (m+2) or four (m+4) ^{13}C atoms in A375 cells transfected with FAH-overexpression or control plasmids for 48 h. Cells were cultured with ^{13}C-D-glucose for 24 h and analyzed by LC-MS. (**E**) A375 cells were transfected with FAH-overexpression or control plasmids for 56 h and ACLY, ACACA, and ACACB mRNAs were quantified using real-time RT-PCR. Results are expressed as mean ± SEM; $n = 3$. $^{*}p < 0.05$; $^{**}p < 0.01$; $^{***}p < 0.001$ (Student's t-test).

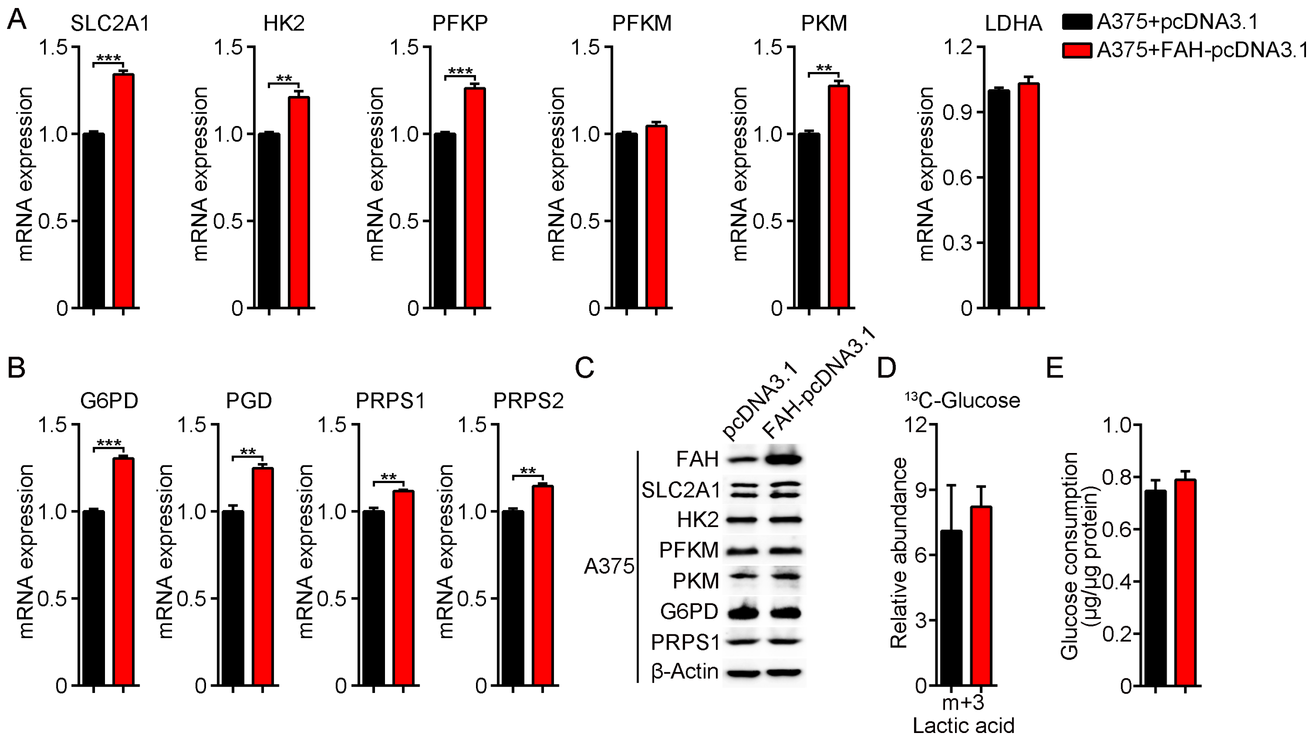

Supplementary Figure 6: Enhanced FAH expression stimulates glycolysis, the pentose phosphate pathway, and nucleotide synthesis in melanoma cells. (**A–B**) A375 cells were transfected with FAH-overexpression or control plasmids for 56 h and SLC2A1, HK2, PFKP, PFKM, PKM, LDHA, G6PD, PGD, PRPS1, and PRPS2 mRNAs were measured using real-time RT-PCR. (**C**) A375 cells were transfected with FAH-overexpression or control plasmids for 72 h and FAH, SLC2A1, HK2, PFKM, PKM, G6PD, and PRPS1 expression was assessed by western blot. (**D**) Relative abundance of lactic acid containing three (m+3) ^{13}C atoms in A375 cells transfected with FAH-overexpression or control plasmids for 48 h. Cells were cultured with ^{13}C-D-glucose for 24 h and analyzed by LC-MS. (**E**) Determination of glucose consumption rate in A375 cells. After transfection with FAH-overexpression or control plasmids for 24 h, measurements were made in cells incubated for 24 h in fresh, complete culture medium. Data are expressed as mean ± SEM; $n = 3$ independent experiments; $^{**}p < 0.01$; $^{***}p < 0.001$ (Student's *t*-test).

Supplementary Table 1: Human melanoma tissue microarray details. See Supplementary_Table_1

Supplementary Table 2: Differentially expressed genes (DEGs) detected by gene microarrays and bioinformatic analysis in A375 cells. See Supplementary_Table_2

Supplementary Table 3: Primer sequences for PCR and siRNAs

Primer sequences for semi-quantitative PCR:

Genes	Forward primers	Reverse primers
FAH(1260bp)	TTGAAGCTTATGTCCTTCATCCCGGTGG	TAACTCGAGTCATGATGGCAGGAGAGCAG

Primer sequences for real-time PCR:

Genes	Forward primers	Reverse primers
FAH (130bp)	TCGGAAGTGTGCATTCATCTC	TCAACGCATTCTCCTTGTCC
β-actin	CCTCGCCTTTGCCGATCC	CGCGGCGATATCATCATCC
CDC5L	CAAGAGGGCTGCAAAGATGG	CGTAACTCCAAGTGAGCCTGT
ACACA	TTCACTCCACCTTGTCAGCGGA	GTCAGAGAAGCAGCCCATCACT
ACACB	GACGAGCTGATCTCCATCCTCA	ATGGACTCCACCTGGTTATGCC
ACLY	AGAAACTGTGGGTCCTTTACTC	CAAGATAGTGTCCAATGAACCC
FH	TGGGAGAATTGATCTTGCCTG	CATGGTCATTGCTTCACACTG
SLC25A1	CTCTGGATGTGATTAAGACCC	CCCTTGTAGAATGCCTTGAG
SLC25A10	TCTTCACTCACTTTGTCGCC	CGAGTCTTCAGCACATCCAG
SLC25A11	TCTCTGACAACATCTTGTGCC	CTTCCCATCAATCATCCGCA
ME1	TCCTCAAGAATGTCTGCCTG	TGCCGTAGTCCAATGTAGAG
SLC2A1	TGGCATCAACGCTGTCTTCT	AGCCAATGGTGGCATACACA
PC	GCCATGTCATGGTAAACGGTCC	GCAGGATGTCTCTGAAACCAGC
G6PD	ACGACGAAGCGCAGACAG	CCGACTGATGGAAGGCATCG
PGD	GAGAAATTGAGACGGTGCCGAG	CACGCTTCTTTGTTCCCTCCT
PRPS1	GGAGACCTGAGTGACCTCCAT	CGGTCCACTTCATTGGCCTT
PRPS2	CAAGGTAGGAGAGAGTCGTGC	ATCTGAGAAGCATGCAGGTCC
HK2	CCCTGCCACCAGACTAAACT	GGATCAGAGCCACAACGTCT
PKM	AATGCAGTCCTGGATGGAGC	CAAGTGGTAGATGGCAGCCT
CS	GCCTGGTATGAGTTGCCCAT	TGACCCATGGAAACAGGAGC
IDH3B	CAAGTCTGTCATCGGTCACCT	GTCTCTTCCCTGGTACACTGC
OGDH	TACTGTGGCGACACTGAAGG	TGGGTTATGTTGGTGAGCGG
DLST	CCAGCAAATGGCGTGATTGA	ACACTGGAGACCTTGACCAC
PFKP	CTATGATGGCTTTGACGGCT	TTGTCCCAAGAATGGAGCCT
PFKM	GATGAGAAGAAATTTGACGAAGCC	TGCGAACCACTCTTAGATACC
PDHB	GCCACAGTTTGGAGTAGGAG	CATAGGGACATCAGCACCAG
LDHA	CGAAGACAAATTGAAGGGAGAG	CGTGATAATGACCAGCTTGGA

Primer sequences for ChIP-qPCR:

Genes	Forward primers	Reverse primers
FAH-15K	AGATTATGTTCATGGGTTTG	AAGGGAGCATTCTGGTTT

siRNAs:

siRNAs	sense	antisense
SiFAH-1	5′ CUACCAUAUGCAAGUCCAA dTdT 3′	3′ dTdT GAUGGUAUACGUUCAGGUU 5′
SiFAH-2	5′ CUACCAACGUCGGAAUCAU dTdT 3′	3′ dTdT GAUGGUUGCAGCCUUAGUA 5′
SiCDC5L-1	5′ CCAGUACACUUUUGUCUGA dTdT3′	5′ UCAGACAAAAGUGUACUGG dTdT3′
SiCDC5L-2	5′ GAACCGCCUUUAACAGAUU dTdT3′	5′ AAUCUGUUAAAGGCGGUUC dTdT3′
